# Parents’ Experiences of Communication With School Nurses About Their Child's Weight Development in Sweden

**DOI:** 10.1177/10598405231221050

**Published:** 2023-12-21

**Authors:** Caroline Skantze, Gerd Almqvist-Tangen, Maria Nyholm, Staffan Karlsson

**Affiliations:** 1 School of Health and Welfare, 3694 Halmstad University, Halmstad, Halland, Sweden; 2 Department of Paediatrics, 3570 University of Gothenburg, Gothenburg, Västra Götaland, Sweden; 3 Faculty of Health Science, 4342 Kristianstad University, Kristianstad, Sweden

**Keywords:** children, communication, parents, school nurses, weight development

## Abstract

This study aimed to describe parents’ experiences of communication with school nurses concerning the growth data and weight development of their children aged 8 and 10 years old in Sweden. Eighteen interviews with parents were conducted and analyzed through qualitative content analysis. The result showed a need for improved dialogue where the parents viewed the health visit's purpose as unclear and lacked feedback*.* The parents desired access to their child's growth data and described the need for an improved channel for receiving information. The parents moreover experienced the lack of a child-centered perspective, described the child's context as not in focus, and desired collaboration around their child. This study shows the need for the development of evidence-based methods for communicating growth data and weight development between School Health Service (SHS) and parents, as well as the need for a reformed SHS perspective towards parents and children.

## Introduction

Nearly one in three school-aged children is living with overweight or obesity according to the WHO European Childhood Obesity Surveillance Initiative (WHO, 2022). A recent study published in Sweden showed an increase in overweight and obesity after the COVID-19 pandemic (Miregård et al., 2023). Childhood obesity increases the risk for Type 2 diabetes, metabolic syndrome, hypertension, dyslipidemia, obstructive sleep apnea, fatty liver as well as obesity as an adult (Jensen et al., 2017; Kumar et al., 2017). Moreover, an elevated body mass index (BMI) as a child has been associated with an increased risk of cardiovascular disease and the development of cancer as an adult (Bendor et al., 2020; Wiehe et al., 2020). Strategies for change in children's dietary and activity levels to prevent overweight and obesity may be effective in making reductions in zBMI scores for children aged 6−12 years (Brown et al., 2019). Prevention of overweight and obesity in childhood is likely to hold major health benefits for the child's future life (WHO, 2022). As these findings indicate that childhood overweight and obesity is a risk factor for later obesity and morbidity, it is important to take advantage of an opportunity for preventive interventions in cooperation with parents.

Parents play a key role in the prevention of childhood overweight, although attention needs to be paid to parental expectations of the feedback from the School Health Service (SHS) (Nnyanzi et al., 2016). Research shows that objective tools such as BMI and growth charts help aid parents’ understanding of weight issues (Bradbury et al., 2018). Communication of the risk of future overweight and obesity to parents was found to be an essential step in childhood obesity prevention. When questioned about prevention, of the onset of overweight in younger children, the parents all described that it was their parental responsibility (Bentley, 2017). The legal aspects of parenthood in Sweden are regulated in the Children and Parents Code (SFS, 1949:381) which declares the responsibilities a parent has as guardian up until the child turns 18 years old, the Convention on the Rights of the Child (SFS, 2018:1197) has also become law in Sweden, thus the best interests of the child shall be a primary consideration. Parents’ need for the necessary information on which to base their decisions concerning their child’s health, is described by school nurses (Skantze et al., 2023). A collaboration between parents and SHS which operate daily wherever children are, may be a feasible way to provide preventive healthcare for school-age children.

Previous research on the prevention of childhood overweight and obesity shows it is a sensitive subject and the communication between parents and school nurses was determined by the parental reactions (Sjunnestrand et al., 2019). Most parents do not perceive their children to have a weight problem, and healthcare professionals need to be aware that parents rarely perceive their children's weight as problematic (Berggren et al., 2018). Parents experienced that children ought to be weighed and measured, however, the issues surrounding healthy growth, especially where there were already concerns about the child’s weight, remained an emotionally loaded topic (Dam et al., 2019). Parents’ experiences of receiving feedback that their child was overweight or very overweight, suggest they considered it a criticism of their parenting skills (Gillison et al., 2014). In a study on a UK-based online forum for parents questioning whether children's weight is a public or private issue, parents experienced legitimate feedback and judgment from health professionals, but they were also concerned with the measurements of children in general (Kovacs et al., 2018). According to a study by Schwartz (2015) concerning BMI notification to parents, parents’ experience of the health screening process was that it was valuable, but the main concern was a lack of communication about and understanding of the process. More knowledge about the parental experience of SHS communicating their children's growth status is thus needed. Accordingly, the aim of the study was to describe the parents’ experiences of communication with school nurses concerning the growth data and weight development of their children aged 8 and 10 years old in Sweden.

## Method

### Study Context

In Sweden, parents have the right and obligation to decide matters relating to the child's personal affairs, for example, health care. However, they must, in line with the child's growing age and development, increasingly consider the child's views and wishes. The SHS should screen and detect deviations in weight, height, or BMI. In case of obesity, the children are referred to a children's clinic. Furthermore, close cooperation between the pupil, the parents, and SHS is essential when the health visits are carried out (The Swedish National Board of Health and Welfare & The National Agency of Education, 2016). Four evenly distributed health visits with measurements such as height, weight, and BMI calculation, are offered by SHS at the ages of six, eight, 10, and 13 years of age. The health visits are not mandatory but offered nationally by the SHS and conducted by the school nurse at the child's school. At the age of 6 years old, the parents are asked to participate in the health visit but not at 8 and 10 years old. At the age of ten, a questionnaire regarding health and lifestyle issues is offered to the parents and the child. Following guidance from the National Board of Health and Welfare and the Swedish Board of Education, the health examinations are recommended to take place on four occasions, however, the reason for this is not specified. Further, there are no standards or routines for notification of health examinations to the parents. The purpose of the health visits is to detect signs of physical and mental unhealth, but risk and protective factors are also evaluated, for example, physical activity, eating, and sleeping habits (The Swedish National Board of Health and Welfare & the National Agency of Education, 2016). Recent National Guidelines for care in case of obesity indicate the importance of respectful care, early interventions, and ensuring that communication with children and parents about health related to weight and obesity is done in a safe and respectful way (The Swedish National Board of Health and Welfare, 2023).

### Participants

The design of the study is descriptive and qualitative. A sample of 18 parents were included via purposive sampling, 15 women and three men (Table 1). The geographical setting of the study was in five different municipalities in south-west Sweden. Inclusion criteria were parents to children that had performed health visits at the age of 8 and 10 years in the SHS, which implied children between 8 and 11 years of age. In discussion with the research group, the 6-year-old children are in preschool and the 13-year-old children have entered puberty, therefore, to get a more homogenous group the children at 8 and 10 years old were included. Approval by the head of administration for children and education in each municipality and the principal for each independent school was obtained. Written information, informed consent, and a request for participation in the study were sent to the parents via each school’s educational IT support, which is used for communication between school staff and parents. Contact by interested parents was made directly to the first author, and the date for the interview was decided over e-mail or telephone.

**Table 1.** Demographic Characteristics of the Parents and Their Children (n = 18).

Characteristics	Number
Sex of the parents	
Female Male Sex of the children	15 3
Female	8
Male	10
Age of the children	
9 years	9
10 years	3
11 years	6

### Data Collection

Data were collected through in-person interviews with all parents which were conducted during September and October 2021 at a place of their choice. The interviews followed a semi-structured interview guide with five open questions and supplementary questions, if needed. The questions were as follows: When your child has had a health visit, can you tell us how you, as a parent, receive information from the school nurse about your child's growth? Would you please describe the information you receive from the school nurse about your child's weight, height, and BMI? How do you as a parent receive an explanation from the school nurse of the meaning of underweight, normal weight, overweight, and obesity? Do you have reflections upon how you would like to receive support from the school health for managing your child's weight development? Do you have reflections upon how a collaboration with the school health would be feasible around your child's weight development? All interviews were conducted by the first author. The length of the interviews varied between 12 and 25 min with a median length of 15 min. The interviews were recorded digitally and then transcribed.

### Analysis

Qualitative content analysis according to Graneheim et al. (2004, 2017), was used to analyze the data. The transcribed interviews were read through several times to acquire an understanding of the content and discussed within the research group. Next, units with a central meaning related to the aim were successively identified in the material and abbreviated into condensed meaning units. The condensed meaning units were then abstracted and labeled with a code that described the content of each meaning unit. To achieve the trustworthiness of the procedure, the codes were reviewed and discussed with all authors and sorted into subcategories and categories, which constitute the manifest content. Examples from the analysis process—with included meaning units, condensed meaning units, codes, subcategories, and categories—are shown in (Table 2).

**Table 2.** Examples of the Analysis Process.

Meaning unit	Condensed meaning unit	Code	Subcategory	Category
But that's the thing with this school nurse, I haven’t quite got control of what her mission really is.	Not fully understood what the school nurse's mission is	Unclearness of the mission of the school nurse	Unclear purpose of the health visit	Needing improved dialogue
No, there has been extremely little information or communication from that part of the school, I would say.	Extremely little information or communication from that part of the school	Extremely little communication	Lacking feedback	
Couldn’t they have a “school nurse site” or information page, where you get the information about your child from the day you are enrolled in school until they leave.	A “school nurse site” or information page about their child from the time they are enrolled until they leave school.	Wanting a “school nurse site” during all the child's school years.	Improved channel for receiving information	Wanting accessibility
No, but what I would like is for the SHS to continue those weight charts that you had from CHC. That the control continued and that it was entered so you had access to it.	The SHS continued with weight charts, and it was entered so you had access to it.	Continue with growth curves and have access to them.	Access to growth data and charts	
Yes, I think it is important to look at how the child lives, how it eats, how it moves, and what joys it has. It is just as important for physical health as for mental health.	It is important to look at how the child lives, for physical health as well as for mental health.	To look at how the child lives is most important	The child's context is not in focus	Lacking a child-centered perspective
A need for more inviting language, and that I would be happy to talk more with you as a parent about this.	Inviting language and that I would be happy to talk with you as a parent.	Would like more inviting language to parents.	Strive for collaboration around the child	

### Ethical Considerations

The study was carried out according to the guidelines laid down in the Helsinki Declaration and approved by the Swedish Ethical Review Authority (Dnr 2020-00567). The informed consent of the participants was obtained prior to each interview.

## Findings

The analysis resulted in three main categories *Needing improved dialogue*, *Wanting accessibility*, and *Lacking* a *child-centered perspective.* These were then divided into six subcategories, reflecting the parents’ experience of communication with school nurses concerning the growth data and weight development of their children aged 8 and 10 years old (Table 3).

**Table 3.** Categories and Subcategories Describing Parents’ Experiences of Communication With School Nurses About Their Child's Weight Development.

Categories	Subcategories
Needing improved dialogue	Unclear purpose of the health visit Lacking feedback
Wanting accessibility	Improved channel for receiving information Access to growth data and charts
Lacking a child-centered perspective	The child's context is not in focus Strive for collaboration around the child

### Needing Improved Dialogue

The parents expressed an unclear understanding of the goal of the health visit and experienced a lack of feedback regarding their child's health visit to the school nurse.

#### Unclear Purpose of the Health Visit

Uncertainty concerning the purpose of the health visit was a recurring parental concern. A lack of explanation to both the child and parents as to why the health visit was being carried out and ambiguity as to what kind of information can be expected after such a health visit is completed. “But that's the thing with this school nurse, I haven’t quite understood what her mission really is” (Interview 10). When they did not receive any notice from the school nurse, the parents presumed all was well with their child. “No, but typically if you don’t hear anything, you assume that everything is fine” (Interview 3). The parents also mentioned having the impression that the school nurse considered it more important to complete the required professional tasks than the care for the children. “Yes, a little counterproductive. What do we want with this? It is as if the nurse should check off her tasks and report that it has been done this year or is there any forward thinking, that it should aim for something positive”? (Interview 17). The parents also expressed the wish that the school nurse conduct more outreach in the work with the children, requesting overall that the SHS to be more active in the school, sports, and outdoor activities.

#### Lacking Feedback

The parents vocalized a pronounced lack of communication and feedback from the school nurse regarding their child's growth. “No, there has been extremely little information or communication regarding that, I would say” (Interview 3). They also experienced that they were not always informed by the school nurse of the health visits, and when informed, only received the minimum information, namely a confirmation that a health visit had been completed. “No, so I generally think that just some kind of indication is better than nothing of course” (Interview 3). As no clear explanation of the concepts of underweight, normal weight, overweight, or obesity was provided by the school nurse, the parents called for feedback regarding their child's growth data after a completed health visit. They described an expectation of further information or confirmation that everything is well with their child. “To be able to get guidelines of what is the normal growth and what is a normal deviation and maybe a bit more about diet and lifestyle and movement, there should come a little more from the school” (Interview 18).

The parents described sending mail to the school nurse on their own initiative when information was not forthcoming. “So, then I wrote to the school nurse and asked how things went and if she doesn’t look at the general condition of the child as well?” (Interview 1). They also explained that they would turn elsewhere to ask for advice. Moreover, if they were worried, the parents searched online for information or contacted a primary care provider or the pediatric clinic. The parents described a clear difference between their child attending child health care (CHC) (0–6 years of age) and SHS “There is a huge difference compared to when the children were smaller, and you went to child health care. Then you got back very clearly, a standard deviation over or under, but I feel that disappears when you enter school” (Interview 18).

### Wanting Accessibility

The parents noted the absence of a channel for receiving information from the school nurse and a preference for a digital solution, as well as access to their child's growth data and charts.

#### Improved Channel for Receiving Information

The parents lacked an established information channel between them, the school nurse, and SHS. They mentioned a personal meeting with the school nurse together with the child once during the first year of school. During the health visit, the school nurse measured the height and weight of the child and showed the parents the growth data. In the following school years, a few parents described their child bringing home a note with current height and weight noted or a paper sent home with the growth data. “In terms of growth, we only received a note sent home, half an A4 printed with the child's name and that the child is x centimeters in length and the weight” (Interview 2). In case of deviation, the growth curve was sent home but when there was no deviation, information was communicated via the child. “When there is no clear deviation, it is more via the child saying that I have been to the school nurse today” (Interview 17). The parents desired an increased exchange of information with the school nurse, preferably via existing digital support for communication with the school. “Couldn’t they have a school nurse site or information page, where you get information about your child from the day they enroll in school until they leave” (Interview 1). The parents expressed the need for the possibility to send messages and receive answers from the school nurse via the existing digital communication support. They found it important that the information followed a standard format and requested a notice even if no specific information about the child was given. “Because no information is also a problem, then you don’t know if there is information that you haven’t had access to. You need to be notified that there is no information, it is also a notification” (Interview 10).

#### Access to Growth Data and Charts

The parents missed not having access to the child’s journal and growth data. They expressed a preference to follow their child's growth visually via the weight, length, and BMI curve, as it was valuable for the parents to see the facts concerning their child “In relation to their own curve and really the most important thing is that the hard facts are sent, preferably with a report on how it relates to the nearest 2−3 previous measurements and the tendency there” (Interview 5). The parents wished for a continuation of the growth curves by the CHC services and described a feeling of losing control. “Yes, that there is a journal or something that we have access to, you first go to child health care and are finally enrolled in the school, then after that you lose your grip on the information” (Interview 1).

### Lacking a Child-Centered Perspective

The parents experienced that the school nurse did not focus on the context and needs of the child and expressed a desire to collaborate around the care of their child.

#### The child's Context is Not in Focus

The parents described the child's context as not being the school nurse's main focus. For example, while there is always an explanation for a growth deviation, the child's health contains so much more than the actual growth, for example, the child's social context. “But it is very valuable to have the opportunity to talk in a free conversation about all parts of the child's development, not only the physical growth or diet or exercise, but the whole of everything, because it is so clearly associated” (Interview 5). The parents described being worried when follow-up questions were not asked at the health visit. They experienced a narrow view of what is right and wrong and that children with normal weight receive praise. The parents highlighted that the children must be able to feel good in their own bodies and understand their own growth. “I think that it is very important that children feel good in their bodies, and that is a really important task for health care” (Interview 12). Furthermore, the parents experienced that a strong relationship between the child and the school nurse was significant. They considered it misplaced when the relationship with the parent, and not the child, was prioritized. Continuity with the school nurse is important, and it should feel safe for the child to make contact with the school nurse “And I don’t need to have a relationship with her or anything, the important thing for me is that she builds a relationship with the children, so they have confidence” (Interview 12). The parents wished that the school nurse would invite the child for a talk, in consultation with the parents. Furthermore, the school nurse would dare to talk to children concerning a reasonable explanation for the weight deviation “Children feel differences clearly, but it must not be dangerous to talk about it. I feel that we don’t talk to the children, but over their heads and behind their backs” (Interview 17).

#### Strive for Collaboration Around the Child

The parents agreed they wanted to be included in the communication concerning their child's health visit, suggesting they should also be invited to join. “I think we, as adults, have to join in and explain” (Interview 2). The school nurses approach varied, some parents experienced that the school nurse was easy to reach, while others did not always feel encouraged to maintain contact. They also expressed the lack of constructive dialogue between them and the school nurse without the child present to avoid increasing the stigma around the child's weight and that the responsibility for the family's eating habits and lifestyle lies with the parents. “But for me as a parent, I already want to establish good habits, good culture, good dialogue and so on long before, and the collaboration with the school health service is an important piece of this puzzle” (Interview 5).

## Discussion

This study explores parental experiences of communication with school nurses concerning the growth data and weight development of their children aged 8 and 10 years old. Three main categories developed through the analysis of the data: *Needing improved dialogue, Wanting accessibility,* and *Lacking a child-centered perspective.*

The communication between parents and school nurses about their child's health visit is flawed. The parents in this study experienced this communication as mostly consisting of unilateral information. Feedback concerning the health visit was lacking yet in demand by the parents as well as communication of how underweight, normal weight, overweight and obesity relate to future health risks for the child. This is in line with a Norwegian study, where parents had clear preferences for how they wanted healthcare providers to interact with them. Furthermore, tailored feedback and follow-up to fit the needs of parents and children was shown to be effective (Ames et al., 2020). The absence of communication could be interpreted by the parents that all is well. At the same time, the parents in this study were left to wonder and want a minimum of confirmation that the health visit had been carried out as well as feedback concerning the growth of their child. A consequence of the lack of dialogue was, parents feeling forced to search for reliable support elsewhere, such as online or at primary care providers. They thus described an experience of being left alone when worried about their child's growth. This finding is in line with an earlier study where parents turned elsewhere for support, such as to other parents, or other healthcare providers (Schwartz et al., 2015). A certain lack of clarity in their understanding of the overall goal and aim of the health visit was expressed by the parents, just as they and their children were unsure of what to expect from the school nurse. According to a Finnish study, parents do not know enough about the school nurses’ work yet desire to be more involved in the cooperation (Mäenpää et al., 2008). This also corresponds to an official report from the Swedish Government concerning cohesive care for children and adolescents (SOU 2021:34), which suggests a lack of responsibility by the SHS as a caregiver. The parents in this study also described a sense that the school nurse was focused on “getting the task done” and considered this more important than the actual communication with them about the growth of their child. This shows a wider communicational challenge regarding the main assignment of SHS and the preventive health work of the school nurse, which needs to be further investigated.

There is no channel for parents to access their child's growth data. The need for an easy reachable way of communication between the school nurse and the parents, preferably via an existing digital platform used by the school was requested by the parents in this study. The parents in this study furthermore expressed a preference to follow their child's growth visually via the weight, length, and BMI curve. A review examining BMI screening programs in different states in the United States shows that, despite the controversial nature of school-based BMI screening programs, objective assessment is a resource for parents that they may not get elsewhere (Ruggieri & Bass, 2015). According to an English study, parents indicated that it was helpful to see the average weight and height of their child and requested information on how to interpret BMI data (Gillison et al., 2014). The parents in this study further described an experience of losing control of their child's growth and development when transferred from CHC to SHS. Digital access to the health journal, length, weight, and BMI charts for school children between 6 to 18 years old is limited in Sweden. According to an official report (SOU 2021:34), communication between different levels of health care does not always function well, which leads to important information about the child not reaching the parent. This shows a need for the development of evidence-based methods for communicating growth data and weight development between SHS and parents.

A child-centered perspective is lacking in the collaboration between parents and school nurses. The parents in this study illustrated that there is always an explanation for growth deviation and that the child's health is about so much more than actual growth. This finding is in line with a Swedish study, where parents defined child health, as much as an emotional and spiritual concept than a physical condition (Almqvist-Tangen et al., 2017). The parents’ experiences in the present study were that the child's entire context should be in focus and not only the growth. In a recent study, where parents’ perception of conversations concerning young children's, weight was explored, conversations reflecting their child's and family's situation were found particularly constructive (Eli et al., 2022). In this study, the parents expressed a desire to be included in the communication surrounding the health visit and to be invited to join their child when it took place. This is in line with another Swedish study, where the parents considered it valuable that they were informed in advance and prepared to discuss growth at the health visit (Sjunnestrand et al., 2019). The parents in this study found continuity with the school nurse important, stating that it should feel safe for the child to initiate contact. This corresponds with Article 3 in the Convention on the Rights of the Child (SFS 2018:1197), where the child's perspective and best interest should be the primary consideration. This highlights the need for the development of a reformed SHS perspective towards the parents and the children. Further research is needed to explore the child's views of communication with SHS.

### Methodological Considerations and Limitations

A challenge concerning the trustworthiness of studies using qualitative content analysis is to make the participants’ voices heard or the researcher's interpretations visible in various parts of the research report (Graneheim et al., 2004, 2017). To ensure the trustworthiness of the study (Lincoln & Guba, 1985), the data were based upon interviews with 18 participant parents of children aged 8 and 10 years. The participant parents came from municipal and independent school settings, in both rural and urban contexts in five different municipalities. Transferability was moreover strengthened by including both female and male parents. This generated an abundance of material for analysis as well as a variety of experiences that strengthened the understanding of parents’ impressions concerning their communication with school nurses. The recruitment of parents involved a purposive sampling that was achieved via each school's educational IT support used for communication between school staff and parents, where interested parents contacted the first author themselves. A possible limitation of the study may be that not all parents were active in the IT support, and the fact that those parents with an interest in the question were also those included may have affected the result. The parents were able to choose the setting of the interview—in their home, at the university, or another place—and during the interview, each parent was asked five semi-structured questions, following the interview guide, with supplementary questions asked when needed, which strengthens the credibility of the study.

To ensure the quality of the data, the interviews were recorded and transcribed verbatim from the interviews. The credibility of the study was strengthened as the procedures of content analysis were carried out following a systematic process (Graneheim et al., 2004, 2017) with examples of the analysis process presented in the study. Units with a central meaning related to the aim were extracted from the transcribed interviews by the first author, and then condensed and labeled with codes. To strengthen the dependability of the process, various codes, subcategories, and categories were discussed by all authors until a consensus was reached. Quotations were used to enhance the credibility of the results. The first author had a preunderstanding of the work as a child health nurse and is thus familiar with the context, and this may have affected the interpretation. This preunderstanding may be of value in seeing the content in its right context, thereby contributing to strengthening the confirmability of the study.

## Conclusion

This study places focus on the parent's experience of the communication between them and the school nurses concerning their child's growth data and weight development. The parents expressed a lack of clarity regarding the overall aim of the health visit and what they and their children can expect from the school nurse. This shows a wider communication challenge regarding the preventive health work of the school nurse and the main assignment of SHS. Furthermore, the parents wanted increased information and feedback from the school nurse and desired access to their child's growth data via a digital channel. This shows the need for the development of evidence-based methods for communicating growth data and weight development between the parents and SHS. The parents also expressed a wish for the school nurse to focus on the child's entire social context and not only the growth. This suggests the need for the development of a child-centered perspective in the communication between the parents and the school nurse, thus further research is needed to explore the child's views of the communication with SHS.

## Abbreviations

BMI: body mass index
CHC: child health care
SHS: school health services.
